# Acknowledgment to Reviewers of *Genes* in 2020

**DOI:** 10.3390/genes12020161

**Published:** 2021-01-25

**Authors:** Genes Editorial Office

**Affiliations:** MDPI AG, St. Alban-Anlage 66, 4052 Basel, Switzerland

Peer review is the driving force of journal development, and reviewers are gatekeepers who ensure that *Genes* maintains its standards for the high quality of its published papers. Thanks to the cooperation of our reviewers, in 2020, the median time to first decision was 15.3 days and the median time to publication was 37 days. The editors would like to express their sincere gratitude to the following reviewers for their precious time and dedication, regardless of whether the papers were finally published:
Aaron, Jean E.Lewis, MoragAbadir, EwardLewis, Sarah JAbcouwer, StevenLi, GangAbd El Wahed, AhmedLi, HaiAbe, IchiroLi, HengAbel, Larry ALi, HongjieAbondio, PaoloLi, HuiAboudehen, KaramLi, JiaAbramyan, JohnLi, Jun (University of Hong Kong)Abreha, Kibrom B.Li, Jun (University of Notre Dame)Abugomaa, AmiraLi, JunhaoAcosta-Herrera, MarialbertLi, MingAdachi, ShunsukeLi, RiqingAdamakis, Ioannis-DimosthenisLi, RuijuanAdams, SolomonLi, ShantaoAdayabalam, BalajeeLi, ShuoAdeyanju, Adedayo OLi, SunanAdhikari, SamirLi, TengAdhikary, TillLi, Wei VivianAdina, Turcu-StiolicaLi, WeihanAdjei-Fremah, SarahLi, XiaogangAdroher, F. JavierLi, XiaojunAdvani, VivekLi, XingAfonnikov, Dimitry ArkadievichLi, XuanAfshari, KhashayarLi, XuehuiAgoro, RafiouLi, Zhi-YongAgrawal, AnshuLiang, ChunAguado, JulioLiang, HaiyingAhamd, KamiLiang, PingAhmadi-Afzadi, MasoudLiangjiang, WangAhmed, ZeeshanLiber, ZlatkoAhn, JaegyoonLiberi, GiordanoAiello, DomenicoLiberles, DavidAirenne, KariLibrado, PabloAjiro, MasahikoLicciardello, ConcettaAkagi, SatoshiLiddell, Jeffrey R.Akhgari, AmirLie Fong, SharonAkimov, Sergey A.Liebman, MichaelAkkari, AnthonyLiehr, ThomasAksenova, Anna YLiguori, GiovannaAkutsu, SilviaLim, Chin YanAl-Ashkar, IbrahimLim, Kee SiangAlberdi, AnttonLin, FanAlbert, EliseLin, Myat T.Albizua, IgorLindblad-Toh, KerstinAlbuquerque, AndréLindemann, StephanAleksandrovych, VeronikaLinder, KeithAl-Elakhdar, AmmarLinderholm, AnnaAleti, GajenderLindholm, AmandaAlexander, Francis Borgio J.Lindholm, MaleneAlexander, MathewLindo, JohnAlexandre, FellousLindquist, Anthea C.Alexandrov, LudmilLing, JiaAlexiadou, ArtemisLing, Xuefeng B.Alfarouk, Khalid OmerLink, Christopher D.Ali, NahidLinton, EricAlicja, Macko-PodgorniLio, DoAlikhan, Nabil-FareedLionikas, ArimantasAliki, XanthopoulouLipkin, EhudAlinikula, JukkaLipson, DavidAlizadeh, MohammadaliLisachov, Artem P.Allain, DanielLista, Florigio RomanoAllen, RandyLitwin, Ireneusz BernardAllikmets, RandoLiu, ChangAllred, Nicholette DanielleLiu, FengAllwood, JuliaLiu, HongxiangAlmahli, HadiaLiu, KunhsiangAl-Mamun, Hawlader AbdullahLiu, ShimingAlmeida, Fausto Bruno Dos ReisLiu, Shing-HwaAlmeida, PedroLiu, ShuyuAlmela, Ramon M.Liu, SiqiAloia, LuigiLiu, WeiAlonso, SantosLiu, YuanAlpar, DonatLlanes, AlejandroAlptekin, BurcuLlorente, BertrandAlqudah, AhmedLo Schiavo, FiorellaAl-Sadi, RanaLo, CliveAlshabib, EbtihalLobocka, MalgorzataAlston, Charlotte LŁobocka, MałgorzataAltintas, Mehmet MLock, AntoniaAl-Tobasei, RafetLoeff, LuukAlvarenga, Danillo OliveiraLoginov, DmitriyAlvarez Trotta, AnnamilLonardi, StefanoAlvarez, AlejandraLondon, Sarah E.Alvarez-Narvaez, SonsirayLongo, VincenzoAlves, CarlosLoosen, Sven H.Amar, DavidLópez Sánchez, AnaAmber, Kyle T.Lopez, Ivan A.Ameele, Jelle Van DenLópez, Jesús AdriánAmini, HajarLópez-Cortegano, EugenioAn, YuŁopieńska-Biernat, ElżbietaAnamthathmakula, PrashanthLorenzis, Gabriella DeAnand, DeeptiLother, AchimAnanga, AnthonyLoturco, Joseph J.Andersen, Ethan J.Lou, ChenguangAnderson, JamesLourenço, AnáliaAnderson, SarahLovrecic, LucaAndongma, Awawing AnjwengwoLow, Siew-KeeAndrade, ArturoLubecka, KatarzynaAndrade, José PedroLucas, TrevorAndrás, GáspárdyLucas-Hahn, AndreaAndras, Jason P.Luchetti, AndreaAndreassen, Rune J.Lucie, GrodeckáAndreotti, GiuseppinaLucienne, VonkAndrés-León, EduardoLukaszewski, Adam J.Andrew, HessLukowitz, WolfgangAndrews, Andrew J.Luna Maldonado, AurelioAndrews, Tallulah S.Lundstrom, KennethAndrzejczuk, SylwiaLungo, Stefano DelAneli, SerenaLupton, Michelle K.Anreiter, InaLussana, FedericoAnsa, BenjaminLuthringer, RemyAnslan, StenLuykx, Jurjen J.Antal, LászlóLuzio, AnaAnton, LennikovLy, PeterAntonacci, RacheleLynch, TimAntonini, MarcoLyons, ShawnAntony, Justin SM’kacher, RadhiaAntunes, MauricioMa, GangAoki, KohMa, ShiningArai, MasamiMa, WanshuAraniti, FabrizioMa, Wen-JuanAraujo, Natalia S.Ma, XiaotuArbeithuber, BarbaraMacdonald, Stuart J.Arbitrio, MariamenaMachida-Hirano, RyokoArgente, María-JoséMachol, KerenArgento, SergioMacholán, MilošArguello, HectorMacia, AngelaArias, AndresMackiewicz, DorotaArisz, StevenMackrill, JohnArmand, Anne-SophieMacLaren, RobertArmengol, RamonMacLeod, IonaArmero Ibáñez, EvaMaconochie, MarkArnaiz, AnaMacQueen, AliceArnholdt-Schmitt, BirgitMaculewicz, EwelinaArnosti, DavidMadarasz, EmiliaArojju, Sai KrishnaMaddamsetti, RohanArroyo Pardo, EduardoMaddox, J. DylanArslan, AhmedMadireddy, AdvaithaArtemaki, Pinelopi I.Madireddy, LohithArtemyeva, AnnaMadrigal, IreneArveiler, BenoitMaduro, MorrisAsai-Coakwell, MikaMaekawa, ShunAseervatham, JayaMafessoni, FabrizioAsensi, VictorMager, JesseAshley, SarahMagli, AdrianoAsimaki, AngelikiMagnaldo, ThierryAsíns, María JoséMagner, MartinAsselin, Sean RobertMahadevan, PadmanabhanAssogba, Benoît SessinouMahalingaiah, PrathapAstin, JonathanMahmoud, MedhatAstuti, Galuh D. N.Mahmud, IqbalAtanasov, VasilMahroo, OmarAttard, AgnèsMaio, Antonia DeAttia, Youssef A.Maj, Mary C.Aubert-Kato, NathanaelMajello, BarbaraAulicino, FrancescoMajumder, PrithaAung, Htin LinMajumder, RinkuAustriaco, NicanorMak, AngelAutexier, ChantalMakarenko, Maxim StanislavovichAvagliano, AngelicaMakino, HiroshiÁvila-Fernández, AlmudenaMakunin, AlexAwate, SanketMakunin, Alexey I.Axiotis, EvangelosMalandrakis, Anastasios AAyon, RamonMalécot, ValéryAzcona San Julian, María CristinaMalfatti, EdoardoAzmi, Asfar SohailMallela, Shamroop KumarAzuma, ChiekoMallo, NataliaBaarends, WillyMalouf, CamilleBaas, PeterMaloy, StanleyBabenko, VladimirMalpeli, GiorgioBabenko, Vladimir NikolaevichMan, ZhouBabiak, IgorManasanch, ElisabetBachmann, HerwigManavalan, BalachandranBaduel, PierreManchia, MirkoBaehr, WolfgangMancini, EmilianoBagyinszky, EvaMandala, Maria TeresaBähr, AndreaManey, DonnaBai, BingMangini, GiacomoBailleul, JustineMango, LucioBainard, JillianManicardi, Gian CarloBairwa, GauravManickavelu, AlaguBajaj, JeevishaManikandan, VeeraBajsa-Hirschel, JoannaMankin, Richard W.Bakloushinskaya, IrinaManzini, ChiaraBakovic, VidManzini, IvanBalaban, Daniel VasileMao, Meng (University of california merced)Balakirev, Evgeniy S.Mao, Meng (University of Georgia)Balcerczak, EwaMaragkakis, EmmanouilBaldo, LauraMarais, GabrielBaldwin, Ransom L.Marec, FrantišekBalestrazzi, AlmaMarek, Laura FredrickBalfagón Sanmartín, DamiánMargaglione, MaurizioBalic, AdamMargaritopoulou, TheoniBallen Taborda, Ana CarolinaMarhadour, SylvieBallini, ElsaMarians, Kenneth J.Balmer, LoisMarimpietri, DaniloBalusamy, Sri RenukadeviMarín, MacarenaBanas, AgnieszkaMarino Gammazza, AntonellaBanerjee, AnweshaMariot, VirginieBanerjee, AparajitaMarketou, Maria E.Bánfalvi, ZsófiaMarkey, Michael P.Banilas, GeorgiosMarkov, GabrielBaptista, Rodrigo De PaulaMarques, Catarina A.Barak, BoazMarques, PedroBaranello, LauraMarrano, AnnaritaBarash, DannyMarsano, René MassimilianoBarba, Cecilio JoséMartella, VitoBarbanera, FilippoMartelli, Pier LuigiBarclay, Sarah F.Martienssen, Robert A.Bardoni, BarbaraMartignago, DamianoBarlow, AxelMartin, Luc J.Barnaby, JinyoungMartin, MargaritaBarnett, BradMartin, MelissaBaro, Jesus A.Martin, TiphaineBarranco-Chamorro, InmaculadaMartín-Burriel, InmaculadaBarrey, EricMartin-Castellanos, CristinaBarría, AgustínMartinez De Toda, FernandoBarrientos-Priego, Alejandro FacundoMartinez Macias, Maria IsabelBarros, Pedro M.Martínez Núñez, Mario AlbertoBarrs, VanessaMartínez-Gómez, PedroBarry, Kathryn HughesMartínez-Laperche, CarolinaBartel, SabineMartin-Galiano, Antonio J.Bartoli, MarcMartiniano, HugoBarton, SamanthaMartin-Lopez, EduardoBaruffini, EnricoMartino, DavidBas B., Oude MunninkMartin-Pena, AlfonsoBasen, MirkoMaruvka, YosiBashinskaya, V. V.Mary, DidierBashir, KhurramMaselli, ValeriaBasler, KonradMasison, Daniel C.Basu, DebaratiMason, ShaunBasu, ReetobrataMasonbrink, RickBasu, SupratimMassimini, MarcellaBateman, BronwynMasuda, IsaoBatini, ChiaraMatei, IrinaBatista, RitaMateo-Bonmatí, EduardoBattlay, PaulMathai, PrinceBaturova, Maria A.Matharu, NavneetBauer, MarioMathe, EwyBauer, StefanMathews, David HBaum, MichaelMathiopoulos, Kostas D.Baumgartner, StefanMatlashewski, GregBaumrucker, CraigMatny, OadiBaxter, BonnieMatos, ManuelaBaymakova, MagdalenaMatos, PauloBayram, OzgurMatos-Maraví, Pável F.Bazakos, ChristosMatowicka-Karna, JoannaBear, ChristineMatson, Steven W.Becker, ClaudeMatsubara, KeikoBecker, JordanMatsuda, YokoBecking, ThomasMatsui, AkihiroBednarczyk, MarekMatsumoto, HirotakaBedre, ReneshMatsuo, RyotaBegcy, KevinMatsushita, KazuyukiBeggs, Andrew DMatthes, AnnemarieBeguelin, WendyMattiucci, SimonettaBehringer, RichardMatulić, MajaBeiki, HamidMátyás, GáborBej, AsimMatzkin, LucianoBekalu, Zelalem EshetuMauhin, WladimirBekheirnia, Mir RezaMaunz, AndreasBelaidi, Abdel AliMauri, GiancarloBelayew, AlexandraMavrogianni, Vasia S.Bellen, Hugo JMawhinney, ThomasBellstedt, DirkMaxim, AurelBelov, Andrey A.May, JaredBenada, JanMayakonda, AnandBenbow, Harriet RMays, HermanBenda, PetrMazin, PavelBen-Dor, ShifraMazón, GérardBenedetti, ElisabettaMazrier, H.Benes, PetrMazzeo, AuroraBenítez, RitaMazzucato, MonicaBennato, FrancescaMbareche, HamzaBenny, JubinaMcCarty, Jack CraneBenson, Matthew DMcCaughan, Geoffrey W.Ben-Yosef, TamarMcCommis, Kyle S.Berardinelli, FrancescoMcCreight, James DonaldBercier, ValerieMcCrone, John T.Berger, FredericMcCulloch, RichardBergey, ChristinaMcDaneld, Tara GBergoglio, ValérieMcDermott, MichaelBergougnoux, AnneMcGrew, MikeBeritognolo, IsaccoMcInerney-Leo, Aideen M.Bernardi, RosaMcIntosh, RobertBernardini, AndreaMcKinney, BrandonBernardino, Raquel L.McLaughlin, JohnBernatchez, JeanMcLemore, Gabrielle LynnBernatík, OndřejMcManus, KirkBerner, DanielMcnally, Francis JBernstein, PaulMcNevin, DennisBerruyer, RomainMcPhee, KevinBertok, SaraMcPherson, BradleyBertolini, MartaMeade, Kieran G.Bertoni, FrancescoMedvecz, MártaBertran, KateriMeerang, MayuraBervillé, AndréMehraj, HasanBesnard, GuillaumeMehta, Anita KuldeepBest, Robert G.Mehta, GunjanBezu, LucilliaMeiri, NoamBhandarkar, NikhilMeldal, Birgit H. M.Bharadwaj, ShivMelis, ClaudiaBhat, SushantMello, AntoniettaBhatia, NehaMelmaiee, KalpalathaBhatnagar, BhavanaMelo-Ferreira, JoséBhattacharya, SomanonMelosik, IwonaBhattarai, GehendraMelrose, JamesBhattarai, KrishnaMenand, BenoîtBhavsar, Amit P.Menda, NaamaBiagioni, AlessioMendes, Marta A.Bicknell, RossMendes-Soares, HelenaBidel, L.P.R.Mendogni, PaoloBidel, LucMendoza, ManuelBieber, KatjaMeng, DongBielecka, MonikaMeng, XiaoliBielinski, Suzette J.Mengoni, AlessioBierne, HeleneMenuz, KarenBiffani, StefanoMeola, GiovanniBiggs, LeahMerah, OthmaneBignardi, ElioMercati, FrancescoBikov, AndrasMerlo, Manuel AlejandroBilal, GhulamMerry, RyanBilic, IvanaMeru, GeoffreyBilichak, AndriyMessersmith, AmyBilleter, Jean-ChristopheMetodiev, Metodi D.Bilska-Kos, AnnaMetzger, JuliaBilyk, Kevin TMetzler, DirkBindschedler, Laurence VeroniqueMeyer, FolkerBingham, RichardMezzelani, AlessandraBiondi, StefaniaMhlanga, Musa M.Birchler, James A.Miao, QiuhongBisanz, JordanMiazzi, Monica MarilenaBiselli, ChiaraMica, EricaBishnupuri, Kumar S.Michel, MaximilianBišová, KateřinaMicheletti, RudiBiswas, SantanuMichenet, AlexisBizimungu, BenoîtMichiue, TatsuoBizzarri, CarlaMiga, KarenBlackmon, HeathMignano, AntoninoBlackwell, MeredithMigocka-Patrzałek, MartaBlackwell, Timothy S.Miguel, CeliaBlader, PatrickMikel, ZaratieguiBlair, IanMilanova, AneliyaBlake, DerekMilc, JustynaBlanco-Lobo, PilarMilenkovic, DraganBlankers, ThomasMiles, Michael F.Blankesteijn, MatthijsMilitello, GiuseppeBlanton, Susan HalloranMillan, TeresaBlasiak, RobertMillar, J. CameronBlau, NenadMiller, Jason RafeBleilevens, ChristianMiller, RobertBlokland, ArjanMillet, Emilie JBloom, KerryMills, Mary KatherineBocedi, AlessioMina, MarcoBochman, Matthew L.Minervini, GiovanniBocianowski, JanMinutti, CarlosBocquet, BéatriceMiquerol, LucileBoczkowska, MajaMiretti, SilviaBody, SimonMironov, AndreyBogado Pascottini, OsvaldoMironova, Victoria V.Bogevik, AndreMirouze, NicolasBogomolnaya, Lydia M.Mishra, Ajay KumarBohannon, KevinMishto, MicheleBohl, DelphineMisra, Rajesh ChandraBoiani, MicheleMiszalski, ZbigniewBojinov, BojinMitash, NilayBoland, C. RichardMitchell, JudyBollepalli, SailalithaMitchell, KierenBonanno, LauraMitra, JoyBond, AndrewMitsuyama, SusumuBöndel, Katharina B.Mittal, SonamBondoc, IonelMiura, IkuoBondy-Denomy, JoeMiura, SayakaBongiorni, SilviaMiyagaki, TomomitsuBonifay, VincentMiyahara, TairaBonin, SerenaMiyamoto, TatsuoBonkovsky, Herbert L.Miyanishi, HiroshiBonnot, TitouanMiyashita, AkinoriBoonen, SusanneMizuo, KeisukeBooth, Kevin T.Mlocicki, DanielBorchel, AndreasMneimneh, SaadBordes, María Dolores LlobatMoawad, AdelBordonaro, MichaelMochida, YoshiyukiBorisova-Mubarakshina, MariaMoertel, ChristopherBørsting, ClausMogensen, Helle SmidtBoruta, TomaszMoglia, CrisitnaBoscari, AlexandreMohamed, Azza H.Bose, DebojitMohamed, Junaith S.Bossa, CeciliaMohammadi, ShabnamBossu, ChristenMohandesan, ElmiraBostan, HamedMohanta, Tapan KumarBoswell, NikkiMohler, VolkerBosze, ZsuzsannaMok, Gi Fay (Geoffrey)Botelho, MonicaMolina-Mora, José ArturoBottillo, IreneMöller, GabrieleBoulé, Jean-BaptisteMöller, MichaelBoulton, KayMolnár, Mária JuditBourguet, ErikaMolotsi, AnnelinBourneuf, EmmanuelleMonaghan, SeanBoussios, StergiosMoncayo, RoyBouwman, AniekMonroe, J GreyBovo, SamueleMonteagudo Ibañez, LuisBowman, DarylMontecucco, AlessandraBrachner, AndreasMonteiro, Cátia Marina MachadoBradley, DanielMontero-Calasanz, Maria Del CarmenBraglia, LucaMontes Resano, MartaBraich, ShivrajMontiel, Eugenia E.Brameshuber, MarioMontiel, GrégoryBrancaccio, MariaritaMoore, Richard C.Branchu, PriscillaMoore, Roger A.Brandl, Christopher J.Moore, Thomas FBrandl, EvaMoraes, CarlosBräsen, Jan HinrichMorales, Víctor AbadBreen, MichaelMoran, OscarBrennan, Adrian C.Morán, PalomaBrennan, ReidMorgan, AnnaBrennenstuhl, HeikoMorgan, ClaireBreton, Timothy S.Morgan, Ethan L.Brezgin, SergeyMorgan, Rhianna K.Bridge, CandiceMorgese, Maria GraziaBrieger, AngelaMori, EmilianoBright, Jo-AnneMorin, MatiasBrinton, JemimaMorisada, NaoyaBrion, MariaMorison, IanBrizola, EveliseMorita, NaokiBrown, Anthony M.C.Morita-Takemura, ShokoBrown, Richard J.P.Moriyama, YuutaBrown, WilliamMorohashi, KengoBrownstein, ZipporaMorpurgo, BenjaminBruce, RobertMorris, KatrinaBrucoli, MatteoMosqueira, MatiasBrugnoni, RaffaellaMosquera, Laura MuiñoBrunet, Bryan M. T.Motoc, Andrei GheorgheBrunet, FrédéricMott, AlexanderBrunoni, FedericaMouhieddine, Tarek H.Bruns, Deborah A.Moulos, PanagiotisBruschi, MarcoMousel, Michelle R.Brychkova, GalinaMousset, SylvainBu, LijingMozaffar, TahseenBubbico, LucianoMozolewska, Magdalena A.Bucci, John P.Mravinac, BrankicaBuchler, Nicolas E.Mrazek, JanBudowle, BruceMrozik, Krzysztof M.Bugno-Poniewierska, MonikaMu, PingBui, Duc-CuongMudge, AgnieszkaBuishand, Floryne O.Mueller-Roeber, BerndBuisson, RémiMuhammad, TahirBujan, JuliaMühlhaus, TimoBukvic, NenadMukherjee, KusumikaBunch, HeeyounMukherjee, RatnadeepBurczyk, JarosławMukherjee, SubhasBurger, PamelaMukhopadhyay, SumanBurmistrz, MichalMukiibi, RobertBurnette, William BryanMullenders, LeonBurow, MarkMuller, JeanBurrack, LauraMuniyan, SakthivelBurrola-Aguilar, CristinaMunshi, KaizadBus, Vincent G.M.Muntané, GerardBush, EliotMunyard, Kylie A.Bush, Stephen J.Murakami, HiroshiBushley, KathrynMurakami, NobuyukiBuszewicz, DanielMurakami, YusukeButali, AzeezMuraro, Mauro JButler, MichaelMuratani, MasafumiButnariu, MonicaMurdoch, BrendaBüttner, SabrinaMurray, Thomas ScotButts, IanMurru, ElisabettaButusov, DenisMurthy Chavali, Venkata RamanaByrd, Alicia K.Musier-Forsyth, KarinCabal Rosel, AdrianaMusolf, AnthonyCaballero, José L.Musselman, Catherine A.Cabeza, Ricardo A.Mustachio, LisaCabiati, ManuelaMustroph, AngelikaCabiscol, ElisaMuthuramu, IlayarajaCabot, Ryan AMutlu, Ayse SenaCadigan, KennethMutshinda, CrispinCadilla, CarmenMuzzalupo, InnocenzoCaeiro, Maria FilomenaMwangi, William N.Cafarelli, AndreaMylonas, IoannisCafforio, PaolaMyskow, BeataCaglayan, MelikeNadal, MarcCahalon, LioraNadeau, Joseph H.Cahill, MeganNader, GustavoCahmayou, SandrineNagai, KeisukeCai, JiyangNagaki, KiyotakaCai, LichunNagamine, YoshitakaCai, WenlongNagarajan, RagupathiCai, YunpengNagashima, YukihiroCalabrese, ChiaraNagel, StefanCalda, PavelNagy, BélaCalderón-Rosete, GabinaNahum, AmitCalla, BernardaNaim, ValeriaCalla, JaesonNakagawa, TakuroCalle-Fabregat, Carlos De LaNakai, HiroyukiCalò, Pietro GiorgioNakaminami, KentaroCalvo, Jorge H.Nakamura, ChiharuCamacho Vallejo, María EsperanzaNakamura, Shun-ichiCamaioni, AntonellaNakayama, MasayoshiCampana, Michael G.Nakazawa, TakehitoCampbell, Karen L.Nan, JiangCampiche, RemoNanni, LauraCampione, MarinaNantasenamat, ChaninCampos Laborie, Francisco JoséNaoumkina, MarinaCampuzano, OscarNarayan, EdwardCANBERK, SuleNarayanan, AnandCancelliere, DanielaNardi, FrancescoCandiani, SimonaNaro-Maciel, EugeniaCândido, ElizabeteNarra, Hema PrasadCang, ZixuanNaryzhny, StanislavCannarella, RossellaNashine, SonaliCano-Espinosa, CarlosNasim, TalatCantalapiedra, Carlos PNath, Vishnu SukumariCao, ChuanhaiNathanailides, CosmasCapellini, Terence D.Nathanson, LubovCaponetti, Gabriel C.Navarro, ElisaCaporali, LeonardoNavarro, EstanisCappelletti, MartinaNavarro, Juan AntonioCaputo, ValerioNavas, AlfonsoCardone, Maria FrancescaNawade, BhagwatCariati, FedericaNde, Pius N.Cariboni, AnnaNdegwa, Eunice N.Carlier, AurélienNedoluzhko, Artem ValerievichCarlon, MarianneNeginskaya, Maria A.Carmeli, YehudaNeji, MohamedCarmo, Fillipe Luiz Rosa DoNeupane, MaheshCaro, TimNeu-Yilik, GabrieleCarone, DawnNewman, JohnCarossino, MarianoNewman, William G.Carpentier, SebastienNg, Jason Liang PinCarr, TonyNguyen, CuongCarracedo, AngelNguyen, Dang H.Carruthers, RossNguyen, Joe TruongCaruso, GianlucaNguyen, Van KhanhCarvajal Moreno, FátimaNianiou-Obeidat, IriniCarvajal, Agustin SolaNicaise, CharlesCarvelli, LuciaNickens, David GCasais, RosaNicolas, EmmanuelleCasamassimi, AmeliaNicole, SophieCasanova, Emily LNicolle, RemyCasanueva, Felipe F.Nicosia, AldoCascella, RaffaellaNieduszynski, Conrad A.Cascone, GiuseppeNielsen, Tue KjærgaardCasella, MichelaNieminen, KaisaCaspar, SylvanNigro, ErsiliaCaspari, ThomasNikfarjam, MehrdadCastanera, RaúlNikolaou, ChristoforosCastel, Stephane E.Nilsson, Björn E.Castella, GemmaNilsson, HenrikCastellano, MarNilsson, MariaCastellano, SergiNina, BrutchCastiglioni, BiancaNinova, MariaCastillo, DanielNiraula, PrakashCastori, MarcoNishiuchi, TakumiCastren, MaijaNishiyama, SoichiroCastro Aviles, AlejandroNissim, SaharCastro, JoanaNitiss, JohnCastroverde, Christian Danve M.Niu, HengyaoCatão, Elisa C. P.Nizzardo, MonicaCatoni, MarcoNogel, MónikaCava, ClaudiaNoguchi, EishiCavaliere, AnnafrancaNogueira-Ferreira, RitaCavazzoni, AndreaNogues, IsabelCavoretto, PaoloNolan, Garry P.Cebey-López, MiriamNolin, SallyĆelepirović, NevenkaNomoto, RyoheiCeliński, KonradNonaka, EtsukoCelis-Regalado, Luis G.Nonami, AtsushiCendron, FilippoNoordam, RaymondČepulienė, RitaNorihito, NakamichiCereda, MatteoNørskov-Lauritsen, NielsČerný, MartinNosenko, TetyanaCerón Madrigal, JuliánNovaes, EvandroCerro, Pablo DelNovák, KarelCésar, Aline Silva MelloNovak, PetrChai, HongyanNovikova, PolinaChaigne, AgatheNovroski, NicoleChaitankar, VijenderNowicki, MarcinChaitanya, Ganta VijayxNowrousian, MinouChakladar, JaideepNozik-Grayck, EvaChakrabarti, RajarshiNuc, KatarzynaChan, Shih-HsuanNunziata, AngelinaChan, Ting-FungNurwidya, FarizChand, VaibhavNute, MichaelChandler, MichaelNyati, Kishan KumarChang, Jia-MingNylen, CarolinaChang, Wen-WeiNyquist, PaulChang, Yao - LungO’Donoghue, PatrickChang, Yu-ChaoObeng, Esther A.Chapman, MarkObruca, StanislavCharbonneau, MichelOcampo Daza, DanielCharette, SteveOcchialini, AlessandraCharlesworth, DeborahOczkowicz, MariaChatrikhi, RakeshOdierna, GaetanoChatterjee, AniruddhaOestergaard, Vibe HallundbækChatterjee, ArkaOffringa, Ite A.Chatterjee, SaswatiOgawa, ShinichiroChaves, InêsOghenekaro, Abbot O.Chavez-Calvillo, GabrielaOgielska, MariaChelmonska-Soyta, AnnaOgrodowicz, PiotrChen, ChiderOgunwobi, Olorunseun OChen, Chien-ChinOh, JaehyeonChen, Chih-ChiehOh, YeonYeeChen, HuiOhkubo, TakeshiChen, KenOhyama, TakujiChen, KetingOjeda, Dario I.E.G.O.A.Chen, LisongOkazaki, YasushiChen, MaoshanOkoń, SylwiaChen, MingOkura, MasatoshiChen, Ming-ShunOkut, HayrettinChen, Shuen-EiOlahova, MonikaChen, Ta-ChingOlasagasti, FelixChen, TaipingOld, Julie M.Chen, Xin JieOłdak, MonikaCheng, JianOldroyd, GilesCheng, LixinOlejniczak, MartaCheng, Min-ChihOliveira, ArlindoCheong, Mi SunOliveira, CarlaCheong, Yong HwaOlivera, Elías R.Cherif, EmiraOlivier Dumonteil, EricCherlin, SvetlanaOlivieri, FabrizioChernova, AlinaOlmo, EttoreChessa, StefaniaOlsen, BirgitteCheung, LeonardOlsen, NancyChevalier, Frederic D.Olson, EricChi, Ya-HuiOmar, TutakhelChia-Jung, ChangOniciuc, Elena AlezandraChiciudean, IuliaOnkokesung, NawapornChien, Yin-HsiuOnzima, RobertChilds, Kevin L.Oota, HirokiChinnappan, MahendranOpperman, Charles HChintalapudi, SumanaOrdidge, MatthewChira, SergiuÖrdög, AttilaChiriac, CeciliaOrellana, EstebanCho, JongkiOrelle, CédricCho, Kwang-SooOrengo, DorcasCho, Won KyongOretskaya, Tatyana S.Cho, Yong-GuOrlacchio, AntonioChockalingam, Sriram P.Orlacchio, ArturoChoi, Jae YoungOrozco-terWengel, PabloChoi, Shing WanOrtigoza-Escobar, Juan DaríoChongmei, DongOsada-Oka, MayukoChopra, RatanOsei-Amponsah, RichardChoudhuri, AvikOstevik, Katherine L.Choudhury, Swarup RoyOstrowski, MaciejChowdhury, Imran HussainOtani, MasahiroChoy, Francis Y.M.Oudemans, PeterChoy, Kwong WaiOulhen, NathalieChriste, CamilleOverall, Karen L.Christensen, ShawnOvesna, JaroslavaChristians, ElisabethOwens, David W.Christmas, MatthewOwens, Gregory L.Chrostek, EwaPaatero, IlkkaChu, Daniel KWPadervinskienė, LinaChu, Dinh ToiPadmanabhan, ShaliniChu, ThinhPaez-Garcia, AnaChu, Tin-ChunPagani, LucaChua, Eng GuanPagin, AdrienChung, Byung MinPagliara, StefanoChung, ChangukPagliardini, LucaChung, HenryPain, BertrandChwialkowska, KarolinaPaino, Eduardo NotivolCianci, AntonioPainter, Jodie N.Ciaudo, ConstancePais, PedroCiccarese, SalvatricePal, DebjaniCiesielski, GrzegorzPal, SangitaCijsouw, TonyPalacios-Gimenez, Octavio ManuelCindrova-Davies, TerezaPalazzo, AntonioCinnamon, YuvalPalencia-Madrid, LeireCiotola, FrancescaPallen, MarkCipriani, AlbertoPalma, DanielaCirillo, EmiliaPalmer, Nathan A.Citek, JindrichPalmirotta, RaffaeleClark, CliffordPalumbi, RobertoClark, EmilyPan, TaoClark, Leigh AnnePan, YoulianClark, MelodyPanda, PreetiClark, Natalie MPandey, Dhananjay K.Clark, SamuelPandolfini, TizianaClaude, JulienPANE, KATIAClifton, NicholasPanga, JaipalCockerton, HelenPantaleo, VitantonioCogo, SusannaPanyushkina, AnnaCohen, James I.Papachristodoulou, AlexandrosCole, Logan W.Papageorgiou, AristotelisColeman, JonathanPapaleo, FrancescoColgan, Donald JPapanikolaou, EleniCollier, AndrewPapini, AlessioCollin, Andrew MPapp, BernadettCollin, Rob W.J.Papp, IstvánColuccia, ElisabettaPaquette, BenoitColunga Biancatelli, Ruben Manuel LucianoParadzik, TinaColussi, SilviaParajuli, SarojConcordet, Jean-PaulPârâu, Liviu G.Condamine, FabienParcells, MarkConn, Jan E.Pareek, Chandra ShekharContina, AndreaParent, Jean-SébastienContreras, Lydia M.Parenti, IlariaContreras-Martel, CarlosParet, ClaudiaConway, MichaelPariasca-Tanaka, JuanCook, LauraParikesit, Arli AdityaCooke, BrianPark, Chi-HunCopeland, NikkiPark, Hee-SooCopeland, William C.Park, Phil JuneCorbett, RyanParodi, BarbaraCorcoran, DavidParolin, CarolaCordero-Casanovas, AlexParrino, VincenzoCorkins, Mark E.Parrotta, ElviraCorpas-López, VictorianoPartridge, CharlynCorrado, GiandomenicoParus, AnnaCorral, JavierParvez, Md MasudCorripio, RaquelParvin, Jeffrey D.Corso, GiovanniPascual, Carmen María GarcíaCorti, ChiaraPasqua, MartinaCortis, PierluigiPassarino, GiuseppeCosme, MarcoPasto, AnnaCosoveanu, AndreeaPastorino, PaoloCosseau, CélinePastor-Villaescusa, BelénCosta, M. Manuela R.Patel, Ankit B.Coste, FranckPatel, NibeditaCostello, JamesPater, Justin A.Coster, Wouter DePatlar, BaharCoto, EliecerPatowary, AshokCouce, María LuzPatrick, MatthewCoufal, NicolePattanaik, SitakantaCousminer, DianaPaudel, Keshav RajCovello, GiuseppinaPaul, ParameswariCovo, ShayPaulin, RoxaneCozzani, EmanuelePautassi, RicardoCozzi, CristinaPawlina, KlaudiaCraig, JonathonPazos, Antonio JuanCraven, MeaganPedemonte, NicolettaCrichton, Robert R.Pehlivan, DavutCristina, Gironi LauraPeiris, DinithiCrnkovic, AnaPelka, PeterCrnkovic, SlavenPellegrini, SandraCroft, LarryPelliccia, FrancaCrona, DanielPelullo, MariaCrozet, PierrePeña Quintana, LuisCubellis, MariaPeña-Llopis, SamuelCuena-Lombraña, AlbaPeñate, WenceslaoCui, HaissiPeng, DuoCumbo, CosimoPenin, AlekseyCurci, Pasquale LucaPennerman, Kayla K.Curic, GoranPennington, Katie L.Cushman, Robert APepłońska, BeataCuyabano, BeatrizPerego, RobertaCvek, UrskaPereira, AdrianoCzaplinski, KevinPereira, LeonorCzarnomska, Sylwia D.Pereira, RuteD’Agostino, DonatoPérez De Castro, AnaD’Amelia, VincenzoPerez Hernandez, DanielD’Angelo, FulvioPérez, SalvadorD’Angelo, MichelePérez-Enciso, MiguelD’Elia, DomenicaPérez-Martín, FernandoDa Ros, LetitiaPérez-Montaño, FranciscoDabrowski, MichalPérez-Sánchez, JaumeDada, ReyadPergament, EugeneDafinca, RuxandraPérilleux, ClaireDaimi, HouriaPerini, FrancescoDal Santo, SilviaPerišić, OgnjenDall, DavidPerkovic, Matea NikolacDamasceno, Jeziel DenerPervouchine, DmitriDamiani, ClaudiaPesovic, JovanDamodaran, SureshPesta, MartinDantec, ChristellePeters, KristianDaras, GerasimosPetersen, JessicaDarbani, BehroozPethick, JamieDarras, SébastienPetitot, Anne-SophieDarst, Burcu F.Petreaca, Ruben CDas, Vallabh E.Petrocheilou, ArgyriDavidson, IritPetrova, MayyaDavie, AndrewPetrulionis, MariusDavies, Kay E.Petrut, BogdanDavis, AdamPettitt, JonathanDavis, MelissaPezzilli, RaffaeleDavis, Richard P.Pfeffer, UlrichDe Almeida, José GuilhermePhan, TungDe Almeida, Roberto FarinaPhilip, RuelensDe Angelis, AnnaPhillips, JulieDe Cárcer, GuillermoPi, LiyaDe Chiara, LetiziaPiasecka, AgnieszkaDe Falco, SandroPiccinin, ElenaDe Felice, ElenaPichon, MaximeDe Freitas Fraga, Hugo PachecoPidon, HélèneDe Galarreta, Jose Ignacio RuizPiepho, Hans-PeterDe La Puente, MaríaPieszka, MarekDe La Rosa, RaulPilc, AndrzejDe Luca, AnastasiaPiles, MiriamDe Marco, PatriziaPilot, MalgorzataDe Miglio, Maria RosariaPiluso, GiulioDe Paolis, AngeloPineault, NicolasDe Re, VallìPinkas, JarosławDe Sousa-Coelho, Ana LuísaPinto, MilenaDe Souza Santos, RobertaPinto-Duarte, AntónioDe Summa, SimonaPiontkivska, HelenDe Tullio, Mario C.Piórkowska, KatarzynaDe Vries, JanPiotrowska, HannaDe, TanimaPipan, BarbaraDearlove, BethanyPiras, Ignazio StefanoDebeljak, NatašaPiris, Miguel ÁngelDecaestecker, EllenPirkmajer, SergejDecker, Jared E.Pirola, YuriDe-Cubas, Aguirre A.Piunti, AndreaDedon, PeterPizzolanti, GiuseppeDegola, FrancescaPjanic, MilosDejeu, JérômePlader, WojciechDejie, WangPlatania, ChiaraDekel, YaronPlohl, MiroslavDel Bianco, MartaPohjoismäki, Jaakko L. O.Del Castillo, IgnacioPöhland, RalfDel Greco M., FabiolaPoirot, MarcDel Vecchio, SilviaPolewczyk, AnnaDelespaul, LucilePoli, AlessandroDell’Edera, DomenicoPolidoros, AlexiosDellaire, GrahamPolimanti, RenatoDellière, SarahPolitano, LuisaDeluca, AdamPoma, AdolfoDemars, JuliePomares, EstherDeMayo, Francesco J.Pomari, ElenaDemidenko, EugenePomraning, KyleDemin, CaiPonzi, EricaDemyda-Peyrás, SebastiánPonzoni, IgnacioDeng, Wen-TaoPoon, Carole L.C.Deniskova, TatianaPoot, MartinDennis, AlicePope, ChadDennis, Michael D.Popeijus, Herman E.Densham, RuthPopova, Alexandra A.Denti, Michela A.Porokhovnik, LevDerous, DavinaPorrini, VanessaDesroches-Castan, AgnèsPortela, MartaDeutsch, Alexanderporter, RichardDevaurs, DidierPostma, Alex V.Devinney, Michael J.Posukh, OlgaDey, NandiniPotaczek, Daniel PDhaenens, Claire-MariePotamias, GeorgeDhar, ArunPotapov, Sergey A.Dhillon, BrahamPotrykus, MartaDhooge, Ingeborg Johanna MariaPoulos, RebeccaDhruba, Saugato RahmanPourová, RadkaDi Dato, ValeriaPourová, Radka KremlíkováDi Maro, AntimoPoutanen, MattiDi Matteo, AntonioPoveda, CristinaDi Mise, AnnaritaPowell, MattDi Stasio, LilianaPoza, Juan JoseDíaz-Sala Galeano, María CarmenPozzi, CarloDibb, Nicholas JPramod, SreepriyaDiCenzo, GeorgePrasad, KasavajhalaDickstein, RebeccaPrasov, LevDieci, GiorgioPrat, SalomeDieser, MarkusPrats, Anne-CatherineDieterich, ChristophPrêle, Cecilia M.Diez, OrlandPrerostova, Mgr. SylvaDiGiandomenico, AnthonyPrice, AdamDijk, AaltPrigge, MichaelDillman, CaseyPrimig, MichaelDiMario, PatrickPristaš, PeterDimri, ManaliProietti, SilviaDinis-Oliveira, Ricardo JorgeProkop, Jeremy WDinkins, RandyPucciarelli, SandraDiretto, GianfrancoPucker, BoasDistl, OttmarPuglia, GiuseppeDivashuk, MikhailPuig, MartaDivella, RosaPuig-Butillé, Joan AntonDobens, Leonard L.Puigdomènech, PereDockery, AdrianPulgar, VictorDoekes, Harmen P.Pullan, Steven T.Dogan, TuncaPupilli, FulvioDolgova, OlgaPuślecki, MateuszDolivo, DavidPyeritz, ReedDolo, VincenzaQian, YanrongDolzblasz, AlicjaQin, TingtingDomen, AndreasQiu, JiandingDomenichini, AliceQuek, CameliaDomingo, ConchaQuesnel-Vallières, MathieuDomínguez-Garrido, ElenaQuignon, PascaleDong, HongxuQuilodrán, ClaudioDong, JieRab, AndrásDong, ShoubinRab, PetrDonnell, David M.Raczkowska, AdriannaDonnelly, Callum GeorgeRadenkovic, MiroslavDonnelly, DeirdreRadoslavov, GeorgiDonze-Reiner, Teresa J.Radu, NicoletaDorado, GabrielRadwan, PawełDorn, AnnikaRaftery, Adrian E.Doroftei, BogdanRaghunandan, MayaDownes, KateRahbé, YvanDoyle, Stephen R.Rahman, HabibDragomir, MihneaRahman, KhaledurDraheim, Roger R.Rahman, Mohammad AlinoorDreger, DaynaRahnavard, GholamaliDreher, TheoRahn-Lee, LilahDrissen, RoyRai, Krishan MohanDrögemüller, CordRai, VineetaDrysdale, ThomasRaikar, Sunil S.Du, ChunguangRajendran, Jagathesh ChandraDu, QianRakov, A. V.Du, YuzheRakovski, CyrilDuan, JialeiRallis, CharalamposDuan, ZhenfengRamadoss, Bharathi RajaDuarte, Vinícius SilvaRamalho Venâncio, Anabela SantoDubinin, Mikhail V.Ramalho-Santos, JoaoDubois, AnnickRamich , OlgaDucluzeau, Anne-LiseRamirez-Peña, EsmeraldaĎudáková, ĽubicaRamírez-Tejero, Jorge AntolínDudley, Jaquelin P.Ramos, CatarinaDuenk, PascalRamos, SebastianDufour, SuzanneRamos-Trujillo, ElenaDugar, GauravRamsay, Joshua P.Dumitru, GabrielaRamsay, Timothy G.Dummer, ReinhardRamsden, Simon C.Duncan, GeorgeRamsey, LauraDundr, MiroslavRand, MatthewDunham, Rex A.Randhawa, ImtiazDunisławska, AleksandraRangappa, Raju BheemanahalliDuran, Daniel PRangnekar, AbhijitDuraturo, FrancescaRanjan, AmareshĎurišová, ĽubaRapizzi, ElenaDutt, ManjulRäsänen, KatjaDuvernell, David D.Rasouli, HasanDüzgünes, NejatRass, UlrichDvořáčková, MartinaRastegar, SepandDwiyanti, Maria StefanieRatajska, MagdalenaDyson, Paul J.Ratan, AakroshDziewit, LukaszRatner, NancyDziewulska, DariaRaudsepp, TerjeDzikiewicz-Krawczyk, AgnieszkaRausch, Jason W.E. Heath, KarenRavanidis, StylianosEadie, LauraRavet, KarlEaswaran, HariharanRay, David A.Eberhard, Monika J.B.Raynaud, CécileEccles, Michael RogerRažná, KatarínaEchenique, VivianaRazzaque, SamsadEckert, ChristianRedden, RobertEdamakanti, ChandrakanthReddy, Sudhir PuttyEdenberg, Howard J.Reddy, UmeshEgbert, Jeremy R.Reddy, VishwanathaEgger, BorisRedei, Eva E.Eglinton, Timothy W.Redekar, NeelamEhrenhofer-Murray, AnnRedina, OlgaEhrenschwender, MartinRedrejo Rodriguez, ModestoEhrhardt, ChristopherRedzej, AdamEiring, AnnaReed, Amy McCartEisenhaber, FrankReed, Kent M.Eisenhofer, RaphaelRehrig, ErinEkwemalor, KingsleyReich, AdamElaswad, AhmedReichle, AlbrechtElderkin, CurtReid, KerryEldridge, Mark D. B.Reid, ScottEl-Gewely, Mohamed RaafatReikvam, HåkonEllegood, JacobReinert, StephanEllestad, LauraReinhardt, Dieter P.Ellis, JohnReis, CarlosEllwood, SimonReis, LindaEl-Mabrouk, NadiaReisert, JohannesElsayed, MansourReisser, CelineElshamy, AbdelsamedReissmann, MonikaElshamy, WaelRelling, MaryEmami, PayamRelógio, AngelaEmberti Gialloreti, LeonardoRemacle, ClaireEndersby, NancyRenau-Morata, BegoñaEndo, MotomuRendo Fornet, FernandoEngler, AnnaRengaraj, DeivendranEoli, MaricaReniere, MichelleErben, EstebanRequena, DavidErger, FlorianRequena, Jose MErgin, VolkanReszka, EdytaEriani, GilbertRey, LuisErjavec, Gordana NedicRey, María-DoloresErzurumluoglu, A. MesutReyes-Centeno, HugoEscalona, Hermes E.Reynolds, Lindsay M.Escoffre, Jean-MichelRhee, Jae-SungEscribano, JulioRhind, NicholasEscudé, ChristopheRiaz, SummairaEspadas Villanueva, IsabelRibani, AnisaEspeli, OlivierRicchi, MatteoEspinoza-Tofalos, AnnaRicci, FrancescaEsposito, AlfonsoRiccioni, GiuliaEsposito, VeronicaRickard, Jessica PaigeEstensoro, ItziarRiddiford, Lynn M.Etxebeste, OierRideout, HardyEuclide, Peter T.Riera-Mestre, AntoniEvangelou, MarinaRiggio, ValentinaEvanno, GuillaumeRimbert, AntoineEverman, ElizabethRimoldi, SimonaEwing, AdamRipoli, CristianExcoffon, KateRitchie, MarylynEyer, Pierre-AndreRivera, Natalia VEynard, Sonia E.Rizzo, MilenaFabbri, Maria ChiaraRoalson, Eric H.Facchin, FedericaRobert, HertelFagoonee, SharmilaRoberts, Angharad MFahlman, Richard P.Roberts, ThomasFairclough, Robert H.Roberts, Wade RFaissner, AndreasRobertson, StephenFalchi, RacheleRobic, AnnieFalfoul, YosraRobin, Arif Hasan KhanFalistocco, EgiziaRobinson, D. AshleyFallini, ClaudiaRobinson, KarlFan, LiuminRobinson, MaxFan, LushengRocco, Florencia DiFang, DavidRocheleau, GhislainFang, FrancisRoddy, Adam B.Fang, Yufeng ‘Francis’Rödelsperger, ChristianFaria, Jorge M.S.Rodin, Andrei S.Farina, LorenzoRodolico, CarmeloFarinha, CarlosRodrigues Santiago, LeandroFarman, MarkRodríguez De Cara, María ÁngelesFarr, Christine J.Rodriguez Martinez, HeribertoFarran, Chadi ElRodríguez, IsabelFarris, Sean P.Rodriguez, LuisFasullo, MichaelRodríguez, Matías MorínFatihah, Hasan Nudin NurRodríguez-Contreras, AdriánFatkhudinov, Timur Kh.Rodríguez-Medina, José R.Faure, GuilhemRodriguez-Revenga, LaiaFaustino, RandolphRodriguez-Vita, JuanFava, ViniciusRoe, Judith L.Faville, MartyRoe, Richard MichaelFazleabas, Asgerally TRoesch, SebastianFedak, GeorgeRoger, LaurélineFeher, TamasRoggenbuck, JenniferFeichtinger, GeorgRohan, Fernando M.Feichtinger, MichaelRoig, Francisco J.Fekete, AgnesRolfo, AlessandroFellers, JohnRomagnoli, CeciliaFellner, MartinRomanenko, Svetlana A.Feltus, AlexRommens, JohannaFerchaud, Anne-LaureRong, ChaoFerguson, GayleRoschke, AnnaFerguson, MoragRosen, JeffreyFeris, Edmond J.Rosli, Hernan G.Ferlazzo, NadiaRoss, IanFernández Martínez, José MaríaRoss, Robert L.Fernández, HelenaRossi, OmarFernandez-Falgueras, AnnaRosso, PamelaFernandez-Funez, PedroRossowska, JoannaFernandez-Rojo, Manuel A.Roszak, PawelFernando, DaniloRoth, MorganeFerree, PatrickRoth, Stephen M.Ferrier, DavidRöttinger, EricFerro, AlfredoRousseau, AudreyFetherman, Eric R.Routhu, Nanda KishoreFicklin, Stephen P.Roux, GeraldineFidalgo, MiguelRoux, IsabelleFiers, MartijnRouzine, IgorFigaj, DonataRovatsos, MichailFigueiredo, CéuRowan, BethFigueroa, Carlos R.Równicki, MarcinFilippidou, SevastiRoy, Swapan KumarFinet, CédricRoyo-Brun, CarolinaFinetti-Sialer, Mariella MatildeRozempolska-Rucińska, IwonaFinlaison, Deborah S.Rozov, Serge M.Fintini, DaniloRubin, MatthewFior, RitaRubio, F. JavierFiore, Maria CarolaRubio-Cabetas, M.J.Firsanov, DenisRubtsov, NikolayFischer, Anthony J.Ruderman, DanFiscon, GiuliaRudrabhatla, SairamFisher, DerekRuiz-Ruano, Francisco J.Fiszer, AgnieszkaRuiz-Sainz, José EnriqueFitches, ElaineRuntuwene, Lucky RonaldFletcher, Jessica L.Ruonala, RailiFlisikowska, TatianaRushworth, CatherineFlórez, Laura V.Russell, MarkFlorez-Rueda, Ana MarcelaRussell, ScottFluhr, RobertRusso, AnnapinaFoissac, SylvainRusso-Ponsaran, Nicole M.Folco, Hernan DiegoRuszkowska-Ciastek, BarbaraFoldager, LeslieRutkowski, RobertFoley, Brian T.Rychlik, BlazejFonseca, PabloRzepnikowska, WeronikaFontana, SimonaSaad, MohamedFontanesi, LucasSaavedra, Juan OrellanaForcina, GiovanniSabaliauskaitė, RasaForero-Castro, MaribelSabater-Muñoz, BeatrizForest, FabienSabetta, WilmaForoutan, AidinSabol, IvanForster, JaniceSacco, JamesForster, PeterSaccone, SalvatoreForsyth, JenniferSaddala, Madhu SudhanaFortin, Jessica S.Sadhu, MeruFotouhi, OmidSadowy, EwaFoucras, GillesSaelao, PerotFragoso, MarianaSafra, NoaFrancesconi, SaraSah, NirnathFrancin-Allami, MathildeSaha, MargaretFrank, KrisztiánSaha, ParnaFranks, Jennifer M.Sahni, Sanjeev K.Franzè, AnnamariaSahu, PranavFraschini, RobertaSaitoh, KenjiFraser, Russell S.Saiz, MariaFraslin, ClemenceSakamoto, AyakoFreddi, LucaSakamoto, KensakuFree, StephenSakamoto, TakuyaFreitag, MichaelSakar, GermánFreking, Bradley A.Saksena, NitinFrelichowski, JamesSalas, Lucas AFreytag, SaskiaSalehzadeh-Yazdi, AliFrezza, ChristianSalentijn, Elma M JFridman, EyalSalilew-Wondim, DessieFriedman, ThomasSalmena, LeonardoFriedrich, JulianeSalmon-Divon, MaliFriesen, Christopher R.Salomaki, Eric D.Fris, MeganSalonen, AnneFritz, GerhardSalopek-Sondi, BrankaFrusciante, SarahSaltzman, WendyFry, Lewis E.Salvadori, SusannaFrydrychova, Radmila CapkovaSalvati, EricaFu, Audrey QiuyanSalvi, ElenaFu, FuyouSalvi, SilviaFuchs, OtaSalzano, EmanuelaFujimoto, AkihiroSalzberg, Steven L.Fujimoto, RyoSamimi-Namin, KavehFujino, KenjiSamira, RozalynneFujita, NaokoSamulski, Richard JudeFukui, KenjiSamwald, MatthiasFurstenau, Tara N.San Mauro, DiegoFurusawa, YukihiroSanchez-Martinez, MelchorFurzer, OliverSandberger-Loua, LauraFuso, AndreaSándor, Attila DFyfe, JohnSandoya, GermánGabrielli, BrianSandstrom, PaulGabut, MathieuSang, HelenGac, PawełSangrithi, MaheshGad, AhmedSan-Miguel, AdrianaGafencu, AncaSanna, DariaGaffo, EnricoSan-Segundo, PedroGaj, ThomasSantander, JavierGajdosik, Martina SrajerSantiago, Araceli E.Galeano, EstebanSantiago, James PatrickGali, HimabinduSantos, RosárioGáll, ZsoltSanyal, MrinmoyGallardo, M. EstherSanz, RebecaGallego-Martinez, AlvaroSapkota, Surya DattaGalletta, Brian J.Saraiva, Raúl G.Galli, FrancescoSardone, RodolfoGalli, VanessaSarkar, ArijitaGallicano, G. IanSarparanta, JaakkoGalstyan, AnahitSarrou, EiriniGama, JoaoSarti, Francesca MariaGambadoro, AlessiaSartori, CristinaGamboa, MaribetSasca, DanielGamper, HowardSassenhagen, IngridGan, PamelaSathiyaraj, SrinivasanGan, PeihengSato, AtsukoGandjbakhch, EstelleSato, MasamitsuGantz, Marie G.Satoh, ShigeruGanzhorn, SethSatta, YokoGaravelli, LiviaSaumitou-Laprade, PierreGarcia De La Serrana, DanielSaura, MaríaGarcía Souto, DanielSauter, MargretGarcia, Anastacia MarieSauvage, ChristopherGarcia, MaricarmenSavaglia, JemmaGarcia, MelissaSavchenko, Andrey A.Garcia, VictorSavoie, Jean-MichelGarcía-Martínez, SantiagoSavojardo, CastrenseGarcia-Oliveira, LuisaSawalha, AmrGarcía-Pérez, JavierSawayama, EitaroGarcía-Ríos, EstéfaniSaxena, SugandhaGarcia-Rubio, RocioSazonova, Margarita A.Gardner, MacScarlett, GarryGardón, Juan CarlosSchaaper, RoelGarganese, FrancescaSchares, GereonGarigliani, MutienScheben, ArminGarino, CristianoScheetz, Todd E.Garnas, JeffScheimann, AnnGarre, VictorianoSchelling, Claude P.Garrido Mesa, JoseSchenk, AlexanderGarry, RobertSchepetov, DimitryGasparis, SebastianSchernthaner, JohannGass, PeterScherthan, HarryGassa, AsmaeSchielen, PeterGaudio, LucianoSchilit, SamGault, ChristineSchilling, SusanneGautam, MukeshSchluth-Bolard, CarolineGauthier, Laura DSchmidt, AnjaGaviraghi, MarcoSchmidt, RyanGawel, DamianSchmitt-Keichinger, CorinneGayà-Vidal, MagdalenaSchmitz, Hans-PeterGazouli, MariaSchmutzler, AndreasGe, JianyeSchneider, PeterGeist, JuergenSchodl, KatharinaGeleta, MulatuSchor, IgnacioGendrel, Anne-ValerieSchrader, AndreaGendron, Fernand-PierreSchreiber, JacobGenereux, Diane P.Schröder, ReinhardGenet, CarineSchulte, DörteGeorgakilas, AlexandrosSchulze, StefanieGeorgiou, Christos D.Schumann, JuliaGera, JosephSchunkert, HeribertGerdol, MarcoSchusser, BenjaminGeremek, MaciejSchusser, Gerald FritzGerring, ZacharySchuster, GadiGershony, LizaSchutz, MichaelGesell, AndreasSchwarz, ErikaGessani, SandraSchwarz, UteGeyer, Mark B.Schwarzbacherová, VieraGhigliotti, LauraSchwind, SebastianGhogare, RishikeshSchwob, EtienneGhosh, ArnabScott, EricaGhosh, SharmilaScott, KieranGiacomello, StefaniaScott, RodneyGiampieri, EnricoSeddon, JenniferGiampietro, PhilipSeeherunvong, TossapornGiarla, Thomas C.Seeman, PavelGiarola, ValentinoSeetharam, ArunGibbs, Adrian JSegami, ShuheiGibert, Jean MichelSeidel, ThorstenGibson, William T.Seike, TaisukeGiese, Arnaud P.J.Seiler, Magdalene J.Gietema, Jourik A.Sekhon, RajandeepGilestro, Giorgio F.Sekulovic, OgnjenGillevet, PatrickSela, HananGillis, AnnikaSelf, James EdwardGils, Janine VanSelim, SamehGimenez, EstelaSellers, Katherine JGimm, OliverSember, AlexandrGindraux, FlorelleSemerci, NihanGinevičienė, ValentinaŠenigl, FilipGino, SarahSeong, Keon MookGiontella, AndreaSeptiningsih, EndangGiordano, CarlaSequí-Canet, José MiguelGiovambattista, GuillermoSergeeva, OlgaGiovannetti, MarcoSeroussi, EyalGipson, TerrySerra, GregorioGiron, Maria CeciliaSerrano, IreneGiudice, JimenaSertorio, MathieuGiudici, Kelly VirecoulonSeth-Smith, Helena M. B.Giunta, SimonaŠevčík, JanGkika, DimitraSevcikova, SabinaGladstein, AriellaSeverson, Aaron F.Glen Jr., W. BaileyShafeekh, MuhammedGlick, Benjamin S.Shah, JyotsnaGlover, AnthonyShah, NeerajGoda, TadahiroShah, VrutantGodler, David EShaikh, Mohamad GuftarGodlewski, JanuszShamblin, BrianGoffredo, ElisaShamieh, SaidGohain, Priyakshee BorpatraShao, PengGokcumen, OmerShapiguzov, AlexeyGokhman, VladimirSharbel, TimothyGola, DamianSharma, Adhikarimayum LakhikumarGoldammer, TomSharma, AnkitaGolenberg, Edward M.Sharma, ArunGolyshina, Olga V.Sharma, AshutoshGomez Garay, AranchaSharma, ManuGómez Gómez, FelipeSharma, Prashant P.Gomez Roldan, Maria VictoriaSharma, SumanaGomez, MariaSharma, SwagatGómez-Barrero, SusanaSharwood, RobertGómez-González, BelénShaw, SophieGómez-Herreros, FernandoShchennikova, AnnaGömöry, DušanShearer, Aiden EliotGonçalves, Vanessa FSheiner, LilachGonçalves, VâniaShekhova, ElenaGondek, MichałShelake, Rahul MahadevGong, Seung PyoShelton, DeliaGong, YanShemirani, Amir-HoushangGong, Yu-NongShen, LishuangGonzalez Prendes, RaynerSheng, YueGonzález, Ana M.Shi, HaifeiGonzález-Hernández, Ana I.Shi, HuwenboGonzález-Recio, OscarShidal, ChrisGonzález-Tizón, Ana M.Shim, Yhong-heeGood, Deborah J.Shin, DonghyukGophna, UriShinde, HarshrajGorbushin, AlexanderShin-Ya, KazuoGotkowska-Płachta, AnnaShiozaki, KazuhiroGoto, TaichiroShipley, JanetGoto, TatsuhikoShiura, HirosukeGoulas, AntonisShmakov, Sergey A.Gourion, BenjaminShockey, JayGrabarek, Beniamin OskarShohat-Ophir, GalitGrabinska, KarionaShu, Shin LaGradinaru, Andrei CristianShusei, SatoGraham, Rachel L.Sibirny, Andrei A.Grahn, Robert A.Sieber, Alisa-NaomiGrahofer, AlexanderSiegel, Paul B.Grapputo, AlessandroSieprawska, ApoloniaGreb-Markiewicz, BeataSijen, TitiaGreen, DeannaSilveira, IsabelGreen, RebeccaSilvestri, GiovanninoGreenwold, Matthew J.Silvestri, GiulianaGreer, JudithSim, ValerieGreer, YoshimiSimões, João Carlos CaetanoGregersen, PerSimões, Maria L.Gregić, MajaSimone, CristianoGregório, MicheleSimons, Andrew M.Gregory, StephenSimonsen, Anna K.Grehl, ClaudiusSimpson, PeterGreither, ThomasSincic, NinoGribskov, MichaelSingh, AbhishekGrieco, FrancescoSingh, AnandGrieshop, KarlSingh, DaljitGriffiths, SimonSingh, HarpalGriffitts, Joel S.Singh, NishaGrilz-Seger, GertrudSingh, RameshGrimaldi, Anna MariaSingh, RatnakarGrimholt, UnniSingh, Rupesh KumarGrimm, ChristianSingh, SukhvinderGripp, KarenSinha Ray, ShresthaGrivet, DelphineSinha, NiharikaGropman, Andrea L.Sinha, RohitaGross, Stephane R.Sini, Maria CristinaGrosse-Brinkhaus, ChristineSinkovic, AndrejaGrosveld, GerardSinn, PatrickGrover, CorrinneSissung, Tristan M.Gruppuso, Philip A.Sivanesan, IyyakkannuGruszczyńska, JoannaSivathanu, VivekGruszecki, TomaszSiwek, MariaGrzebelus, DariuszSjöling, ÅsaGrzegorczyk, IzabelaSkalicky, MilanGrzegorzewski, WaldemarSkarzynska, MagdalenaGsaller, FabioSkorko-Glonek, JoannaGu, XiaolianSkorupski, JakubGuallar, DianaSkotak, MaciejGuan, YutingSkovgaard, OleGuardone, LisaŠkrabišová, MáriaGuarnaccia, VladimiroSkrzypski, MarekGubala, AnetaSkuratovskaia, DariaGudys, AdamSkurikhin, Evgenii GermanovichGuertin, David A.Ślaska, BrygidaGuevara, GovindaSlattery, DavidGugel, IsabelSławińska, AnnaGuglielmo, PriscillaSleigh, JamesGugnoni, MilaSlotman, Michel AndreGuillen Fretes, Rosa MariaSmith, Alexander J.Gullì, MariolinaSmith, LeoGully, DjamelSmith, MelanieGundorova, PolinaSmith, RichardGünes, CagataySmolander, Olli-PekkaGünther, AndreasSnead, MartinGünzel, DorotheeSnowdon, RodGuo, GraceSoares, Ana RaquelGuo, Hao-BoSoberón-Chávez, GloriaGuo, YanSobhani, NavidGupta, KuldeepSobinoff, AlexanderGupta, MohanSochaj-Gregorczyk, Alicja MariaGupta, ParulSofou, KalliopiGupta, Radhey S.Soglia, DomingaGurden, HiracSolomou, AlexandraGurgul, ArturSolovchenko, AlexeiGurskaya, Nadya G.Soltabayeva, AigerimGurtler, VolkerSomma, Maria PatriziaGustafson, Dan L.Somo, MohamedGutierrez, ClaudeSoncin, FrancescaGutierrez-Camino, AngelaSong, HaiweiGutsaeva, DianaSONG, JIA L.Guynet, CatherineSong, JiangningGuyot, RomainSong, JiuzhouGuzzi, FrancescoSong, LiujiangGysi, DeisySood, RamanHa, Se EunSoontornniyomkij, VirawudhHa, Yun-SokSordino, PaoloHaan, Peter DeSørensen, MeganHacker, UlrichSotiriou, Sotirios K.Hadi, SibteSoucek, PavelHagerman, Randi J.Soucy, ShannonHagi, TatsuroSouthgate, LauraHagiwara, DaisukeSoveral, GraçaHahn, AndreaSoyano, TakashiHahn, MatthiasŠpakulová, MartaHains, DavidSpäth, Gerald F.Hála, MichalSpengeler-Frischknecht, MirjamHalaban, RuthSpiegelberg, DianaHaley, Bradd JosephSpiegler, VerenaHaley, Nicholas J.Spies, Ingrid BrigetteHalford, StephanieSpínola, HélderHall, LynseySpletter, MariaHall, MichaelSpradling, AllanHall, RebeccaSprecher, SimonHall, RobynSquire, Sylvia AfriyieHallerman, EricSripathy, SmithaHallsworth, J.E.Srivastava, AbhishekHalo, MarkoStacey, DavidHan, DohyunStädler, ThomasHan, Jae YongStaiger, DorotheeHan, MiraŠtajner, NatašaHan, YanStange, KatjaHanau, StefaniaStankevičius, VaidotasHanna, Lauren L HulsmanStanton, BenjaminHanna, NadineStarr-Moss, AlisonHannibal, LucianaStasiak, MagdalenaHansen, AllisonStaskawicz, BrianHappel, Christine M.Stasyk, TarasHarashima, NanaeStavoe, Andrea K.Hare, Matthew P.Steele, John WHarold, DeniseSteensma, Matt R.Harris, StephenStefaniuk-Szmukier, MonikaHarrison, PatrickSteinberg, JuliaHartman, John L.Stella, AlessandroHartman, MariuszStenzel, Tomasz A.Haruta, MiyoshiStępień, ŁukaszHaselkorn, TamaraSterken, MarkHashimi, HassanSterniša, MetaHassani, Mohamed-AmineStevens, David ColeHaubold, BernhardStevens, RebeccaHauser, Alexander SebastianStevenson, WilliamHauser, PhilippeStewart, Cameron R.Hausner, GeorgStieger, KnutHawkins, Christine J.Stocker, HugoHayashi, ShinichiroStöger, TobiasHaynes, ColeStoicanescu, DorinaHayward, Jessica J.Stojak, JoannaHazen, Samuel P.Stolle, EckartHazzouri, Khaled MichelStoltzfus, JonathanHealey, AdamStopnisek, NejcHédan, BenoitStorlazzi, AuroraHegde, Rashmi S.Stougaard, JensHegde, ShivanandŠtrac, Dubravka ŠvobHeifetz, Eli MStracker, TravisHeigwer, FlorianStrain, GeorgeHeilbronner, UrsStrauss, Jerome F. IIIHeitkam, TonyStrelnikov, Vladimir V.Helliwell, ChrisStricker, SigmarHelmy, Yosra A.Stromberg, ZacharyHemberg, MartinStrong, TheresaHemery, LenaigStrong, Theresa V.Henderson, IanStrzala, TomaszHendrickson, TamaraStuart, Rhona K.Hennigs, Jan K.Stuppia, LiborioHenriques, Adriano O.Sturtzel, CaterinaHenriques, RuiSu, YuanjieHenry, RobertSuarez-Vega, AroaHérault, YannSuazo, JoséHerbet, MariolaSubirana, JuanHerbig, AlexanderSuddala, Krishna ChaitanyaHerczeg, DávidSuderman, MatthewHermand, DamienSuhail, YasirHermida, MargaridaSułkowska, MałgorzataHernandez, JesusSullivan, Lori S.Hernández-Ramírez, Laura CSummer, AndreaHerniter, IraSun, DongboHerrero, JavierSun, FengjieHesami, MohsenSun, XuepengHeuermann, MarcSun, ZhengwangHickey, Michael J.Sundheim, LeifHicks, James KevinSuñer, Damian HeineHide, MichihiroSur, SubhayanHiggins, DesSurmacz, LilianaHill, TomSurówka, EwaHindmarch, CharlieSuryavanshi, Santosh V.Hinojosa, LeonardoSuryawanshi, VasantikaHirayama, TakashiSuter, LéonieHirono, KeiichiSuzuki, HitoshiHirsch, IvanSuzuki, IkuoHitachi, KeisukeSuzuki, MasakoHitte, ChristopheSuzuki, MasatakaHizel, CandanSvartman, MartaHjelmen, Carl E.Sverrisdóttir, ElsaHjortshøj, Rasmus L.Swaggerty, ChristinaHo, Shin-LonSwartz, Steven ZacharyHoang, Nam V.Świtoński, MarekHoareau, ThierrySyeda, AishaHocquette, Jean FrancoisSymington, LorraineHoefsloot, E.H.Symonova, RadkaHoffman, Joseph I.Szatkiewicz, Jin P.Hoffmann, Lucia VieiraSzczepanek, JoannaHofreiter, MichaelSzebeni, Gábor JánosHoksza, DavidSzepesi, AgnesHolland, TonySzkuta, BiancaHolopainen, SampsaSznajder, LukaszHonda-Okubo, YoshikazuSztal, TamarHonka, JohannaSzuhai, KarolyHooper, DanielSzymanski, ChristineHoppler, StefanSzymański, ŁukaszHörandl, ElviraSzymański, Maciej IejHorike, Shin-ichiSzymczyk, PiotrHorin, PetrSzyndler-Nędza, MagdalenaHormozdiari, FereydounTabęcka-Łonczyńska, AnnaHorowitz, Abraham RamiTadeo, Francisco R.Horsberg, Tor EinarTae-Jin, KimHoudt, Rob VanTaghizadeh, HosseinHousset, ChantalTagliabracci, AdrianoHouston, BrendanTai, PhillipHouweling, PeterTajeddine, NicolasHowe, Kathryn L.Takabayashi, ShujiHow-Kit, AlexandreTakagi, MasatoshiHozawa, MikaTakahashi, HidekazuHrckulak, DusanTakahashi, KeitaHřibová, EvaTakahashi, TakuHsiao, Kuei-YangTakata, KeiichiHsiao, Shu-HueiTakiya, ShigeharuHsieh, Jui-HuaTalati, ChetasiHsu, Shu-haoTaliercio, EarlHsu, Te-HuaTalukder, ShyamalHu, MinTambe, MitaliHu, ShuiyingTamir, Sharon OvnatHu, YanmeiTamura, KojiHu, ZhenbinTan, Bee KHuang, Hsiao-YunTan, JiafuHuang, Jen-PanTan, YuqiHuang, LingTan, YutingHuang, Shar Yin NaomiTanabe, HideyukiHuang, Shu-PinTanabe, KatsuyukiHuang, TonyTanaka, HisashiHuang, WeihuaTanaka, KozoHuang, XiaoTang, DamuHubal, MonicaTang, KevinHubková, BeátaTangherlini, MichaelHublitz, PhilipTangherloni, AndreaHudson, Amanda L.Tani, MotohiroHuff, David R.Tani, NaokiHugo, Eric R.Taniguchi, HiroakiHuh, Jae-WonTaniguchi, MasaakiHumbolt, PatriceTanurdzic, MilosHummitzsch, KatjaTao, Yong-fuHumphreys, DavidTaranto, FrancescaHung, Shih-YaTarone, AaronHunt, HarrietTavakoli, JavadHunt, Kristopher A.Tavares, ErikaHurst, AnnaTay, Ye DeeHuth, SebastianTaylan, FulyaHvidsten, Torgeir R.Taylor, Erika A.Hwang, Ming-JingTaylor, IsaiahHyeonso, JiTaylor, Kira C.Hyink, Deborah PinsonTaylor, Molly A.Hynes, Michael F.Te Pas, MarinusHynes, NancyTeh, Soon LiHyun, YoubongTeixeira, RitaIacomino, GiuseppeTekmal, Rajeshwar R.Iacopino, SergioTelugu, BhanuIbrahim, AhmedTembrock, Luke R.Ichida, FukikoTempleton, Matthew D.Idili, AndreaTempone, AntonioIdnurm, AlexanderTemsch, EvaIgartua, ErnestoTeng, ShaoleiIglesias-Osma, Maria CarmenTenorio, JairIhnatko, RobertTerada, TomoyoshiIhrie, RebeccaTerai, YoheiIkeda, YoshihisaTerlizzi, VitoIlieva, NevenaTerol, JavierIlinskiy, YuriyTeruel-Montoya, RaúlImamura, FumiakiTesfaye, DawitImbert-Fernandez, YoannisTessema, Gezahegn GirmaImbimbo, BrunoTeteloshvili, NatoImran, Qari MuhammadTevosian, Sergei G.Imumorin, Ikhide G.Thakur, ShilpaIngerslev, Lars RoedThalinger, BettinaInglada-Pérez, LucíaThanigaimani, ShivshankarInoue, TatsuyaThankam, Finosh G.Iorizzo, MassimoThedieck, KathrinIrimia, ManuelThekkiniath, JoseIrish, SethThil Smith, Adam AlexanderIrpino, AntonioThomas, Gregg W.C.Isaacs, William B.Thomas, MiltonIsayenkov, StanislavThomas, PaulIshibashi, KenichiThompson, AndrewIshida, YasukoThompson, RuthIshidate, TakaoThompson, VickiIshii, TetsuyaThongprayoon, CharatIshikawa, AkiraThornell, Ian M.Ishikawa, RyujiTian, RenmaoIshorst, NinaTian, XiuchunIsmaiel, AdnanTieman, Denise MIto, HisashiTierney, Keith B.Ito, NaokiTierney, SimonIto, TakuhiroTilocca, BrunoIvancevic, Atma M.Timmis, JeremyIvankovic, AnteTimoshevskiy, Vladimir A.Ivashchenko, DmitriyTiret, LaurentIvetic, VladanTisné, SébastienIvics, ZoltánTlemsani, CamilleJ. Van Dijk, PeterTodd, LaurenJ. Wing, HelenToffolatti, Silvia LauraJablonowski, ZbigniewToiyama, YujiJackson, MichaelTokarska, MałgorzataJackson, RubieTollis, MarcJacobo-Velazquez, DanielTomasetti, MarcoJacobsen, EvertTomaszkiewicz, MartaJacobsen, Hans-JörgTomé, StéphanieJadhav, ShyamalagauriTomilin, Alexey N.Jaggavarapu, SiddharthTomljanović, TeaJaiswal, DeepikaTonelli, FrancescoJakočiūnas, TadasTong, MingmingJalali, Beenu MozaTonhajzerova, IngridJalli, MarjaTonini, RaffaellaJames, Euan KevinTonkyn, DaveJammes, HélèneToriello, Helga V.Jamshidi, YaldaTornillo, GiusyJancovich, JamesTorrens-Spence, Michael P.Janczar, SzymonTorres Romero, Anam M.Janczarek, MonikaTorres, JasonJaniak, MareikeTosi, SabrinaJankauskiené, AugustinaTozaki, TeruakiJankowicz-Cieslak, Joanna BeataTozzo, PamelaJanni, MichelaTranebjærg, LisbethJanovska, DagmarTraut, WaltherJanssen, EmielTrcek, JanjaJanusz, GrzegorzTreff, Nathan R.Jay, Patrick Y.Trehin, ChristopheJayakodi, MurukarthickTremblay, NicolasJayanthi, SankarTrepanier, AngelaJean-Quartier, ClaireTresch, AchimJensen, Roy A.Trevisson, EvaJeon, Ji-HyeonTrichet, ValérieJernigan, RobertTrifonov, VladimirJetybayev, Ilyas YerkinovichTripp-Valdez, Miguel A.Jhanwar-Uniyal, MeenaTroan, BrigidJimenez, Patricia TTroedsson, Mats H.T.Jin, Sheng ChihTrojner-Bregar, AndrejaJin, YuefeiTrombetta, BeniaminoJo, Yeong DeukTruong, ThereseJohannesson, KerstinTrusca, Georgeta VioletaJohansson, KristinaTsai, KateJohler, SophiaTsarouhas, VasiliosJohnson, JoshuaTseng, Ruo-ChiaJohnson, Sheri L.Tsepilov, YakovJohnsson, MartinTsoi, StephenJondeau, GuillaumeTsubota, TakuyaJones, MarjorieTsukahara, ToshifumiJones, Simon WTsukamoto, DaisukeJones, Wendell D.Tsuzuki, MasayukiJonkers, Iris H.Tuffery-Giraud, SylvieJordan, BrianTulay, PinarJordan, HeatherTuller, TamirJores, JörgTuorto, FrancescaJorge, J. C. LuisTurnbull, Matthew W.Jose, RodriguezTurunen, Mikko P.Joseph, LeoTvedte, Eric SJoseph, SusanTyagi, SonikaJournot, LaurentTylki-Szymańska, AnnaJQ, BoedickerTzen, Jason T.C.Juan Carlos, Espinosa MartínUdroiu, IonJuárez, ManuelUebbing, SeverinJucan, DenisaUetz, PeterJulius, UlrichUhde-Stone, ClaudiaJung, InukUhl, GeorgeJung, Ki-HongUlańczyk, ZofiaJung, KlausUlicna, LiviaJunker Mentzel, Caroline M.Unmack, PeterJunkiert-Czarnecka, AnnaUpadhyay, RakeshJuracek, JaroslavUptmoor, RalfJuranic, MartinaUpton, KyleJust, Katja S.Urbanska, Edyta MariaJust, SteffenUrgese, GianvitoKaczmarek-Ryś, MartaUribe, Juan EstebanKadam, SuhasUsami, Shin-ichiKaesmann, LukasUsher, JaneKagami, MasayoUsmani, AbulKagey, Jacob DUtzeri, Valerio JoeKai, TangUwimana, BrigitteKai, Wang (Children’s hospital of Philadelphia)Uziel, OritKai, Wang (Institute for Systems Biology)V. Peñalba, JoshuaKaiser, AnnetteVaidotas, StankevičiusKajiya-Kanegae, HiromiValasi, IreneKakoi, HironagaValdés-Baizabal, CatalinaKakrana, AtulValencia, JarisKalaitzis, PanagiotisValente, Bruno DouradoKale, SandipValentincic, Natasa VidovicKalemba, Ewa M.Valkai, IldikóKalendar, RuslanVallespin, ElenaKaleta, BeataVan Cutsem, PierreKALIA, AwdheshVan De Laar, IngridKalita, MichałVan Der Velden, Vincent H.J.Kalitsis, PaulVan Eys, Guillaume J.Kallam, KalyaniVan Galen, PeterKalra, DivyaVan Gisbergen, Marike W.Kalyandurg, Pruthvi BalachandraVan Heusden, Adriaan WKaminska, BozenaVan Loo, MarcelaKammenga, Jan E.Van Muiswinkel, Willem B.Kampmann, Marie-LouiseVan Oers, NicolaiKanata, EiriniVan Otterloo, EricKandpal, ManojVan Parys, AlexanderKaneda, MasahiroVan Wezel, GillesKanekatsu, MotokiVan Wuytswinkel, OlivierKaneko, GenVan Wyk, NielKang, CongBaoVanek, DanielKang, HuiminVanselow, JensKang, Ji HyounVanWallendael, AcerKang, YunVaquero Lorenzo, ConcepciónKankare, MaariaVarela, JoãoKanugula, Anantha KoteswararaoVarma, ParulKappel, ChristianVasina, Daria V.Kar, AdwitiyaVassetzky, Yegor S.Karaffova, VieraVatanshenassan, MansourehKaragkouni, DimitraVats, PankajKardailsky, IgorVattipally, SreenuKarki, Hari SharanVaughan, MelKarlov, GennadyVeale, AndrewKarpukhin, Alexander VasilievichVeerabagu, ManikandanKarras, GeorgiosVeillet, FlorianKarre, ShaileshVelazquez, MiguelKasai, FumioVelazquez, RamonKasashima, SatomiVeldhuizen, Ruud A. W.Kase, SatoruVelentzas, Athanassios D.Kashi, YechezkelVeletza, StavroulaKashina, AnnaVeley, Kira M.Kashtalap, VasilyVélez, Ana MaríaKasprzak, AldonaVelsko, IrinaKaspy, AntonyVenkatesan, ThiagarajanKassai, HidetoshiVenkatesh, JelliKastaniotis, Alexander J.Venner, EricKasuga, TakaoVentosa, AntonioKatarzyńska-Banasik, DorotaVera-Gargallo, BlancaKato, YasuhikoVerboom, Diana M.Kato, YoshioVerde, GaetanoKatsavos, SerafeimVerdeguer, FranciscoKatsiotis, AndreasVerdu, PaulKatsuta, ErikoVergadi, EleniKaushik, SwatiVerhagen, IreneKausrud, KyrreVerma, SurbhiKaviani, MinaVerma, VikashKawaguchi, NanakoVermeij, Wilbert P.Kawakatsu, TaijiVerri, CarlaKawamura, KouichiVersacci, PaoloKawaoka, AkiyoshiVerta, Jukka-PekkaKawauchi, KeikoVervelde, LonnekeKayadjanian, NathalieViana, FlaviaKazdal, DanielVicens, AlbertoKazushi, AotoVicient, CarlosKearsey, StephenVickstrom, Casey RKeel, BrittneyVidigal, Joana A.Keersmaecker, Sigrid CJ DeVidovic, SinisaKeil, KimberlyVieira, AlexandreKeinath, MelissaVilas, Julia SanchezKeller, LanceVilla, ChiaraKelly, LauraVillano, CliziaKelm, Robert J.Villaseñor, AlmaKelman, ZviVillaverde, GonzaloKember, RachelVimberg, VladimirKenji, ToyotaVincent-Monégat, CaroleKenny, Nathan JamesVingiani, Giorgio MariaKeogh, KateVingron, MartinKern, RamonaVirdi, KamaldeepKerr, Stephanie C.Viscomi, CarloKerwin, Rachel E.Visscher, AnneKhaliluev, Marat R.Vita, RobertoKhan, Mohammed MoshahidVitale, AlessandroKhan, Saleem AVitart, VeroniqueKhan, ShahanshahVitte, ClémentineKhanal, SameerVittori, MilošKhanh, Tran DangVives Cobo, CristinaKhanim, FarhatVizoso-Vázquez, AngelKhansari, Ali RezaVladimirov, VladimirKheimar, Ahmed M.Vlaykova, TatyanaKhivansara, VishalVo, NguyenKhoeiry, NathalieVoelkers, MirkoKhomtchouk, BohdanVogg, Matthias ChristianKidd, Jeffrey M.Vogl, ClausKidd, Kenneth K.Voigt, KerstinKidd, ThomasVolleth, MarianneKido, TatsuoVona, BarbaraKidron, HeidiVoncken, Jan WillemKiełbowicz-Matuk, AgnieszkaVoronova, AngelikaKiesler, Kevin M.Voskarides, KonstantinosKikuchi, TaiseiVossaert, LiesbethKil, Eui-JoonVozdová, MilušeKilár, FerencVozhzhova, Nataliya N.Kim, Hyun HeeVrettos, NicholasKim, HyungVujosevic, MladenKim, Jee TaekVukmirovic, MilicaKim, Jong GugWachowska, UrszulaKim, JongminWagner, DominiqueKim, JunilWagner, GunterKim, JunmoWagner, JasenkaKim, KyoungtaeWagner, Kay DietrichKim, Lark KyunWahi, KanuKim, ManhoWaidmann, SaschaKim, NamshinWakamatsu, NobuakiKim, Raymond HWalker, Graham C.Kim, Sang BumWalker, Joseph F.Kim, Sang-TaeWallace, Ian S.Kim, SungeunWallis, DeeannKim, Young RanWalsh, JenniferKim, YoungjoWalsh, RoddyKimbrel, JeffreyWalter, Michael A.Kimeklis, Anastasiia K.Walters, James R.King, TuriWalters, Julian R. F.King, W. AllanWang, Bang-AnKingsley, RobWang, BoKirac, IvaWang, ChaoKirchberger, PaulWang, ChunKirchmaier, AnnWang, Hailin Kirczuk, LucynaWang, JinkaiKirk, DevinWang, LingfeiKirk, Edwin P.Wang, MaosenKirkbride, RyanWang, MingKirsebom, LeifWang, PengchengKirtiklis, LechWang, PenghaoKishita, YoshihitoWang, ShikeKisker, CarolineWang, ShixiongKitazawa, SoheiWang, ShizhiKizis, DimosthenisWang, XiaodongKjos, MortenWang, XuKlassen, RolandWang, XuewenKlčová, LenkaWang, YanchangKlein, Arnaud FWANG, YUNG-SONGKlein-Seetharaman, JudithWang, ZhaoningKlepikova, Anna V.Wang, ZhengKloch, AgnieszkaWanrooij, PaulinaKloosterman, AteWarchoł, MarzenaKlug, DennisWard, DouglasKlymiuk, ValentynaWard, JodieKmiec, Eric B.Warnow, TandyKnapczyk-Stwora, KatarzynaWarren, Helen RKnijnenburg, JeroenWasylishen, AmandaKnytl, MartinWatanabe, Kazuo N.Ko, Dae KwanWatanabe, Yuichiro (Niigata University)Koba, TakatoWatanabe, Yuichiro (University of Tokyo)Kobayashi, MakotoWatson, DionysiosKodedová, MarieWatson, SarahKodidela, SunithaWcisel, DustinKoestler, Benjamin JWebb, AmyKofler, RobertWebber, CalebKoga, HiroyukiWeigel, Ronald J.Kogelman, LisetteWein, AlexanderKohan-Ghadr, Hamid-RezaWeis, Allison M.Köhn, MarcelWeiser, Brian P.Koide, TetsuyaWeiss, JuliaKolaczkowski, BryanWeissensteiner, Matthias H.Kolář, MichalWelch, CarrieKolbe, DianaWellband, KyleKole, ChristoWen, JiaKolenda, MagdalenaWende, WolfgangKolling, StefanWendel, JonathanKolora, RohitWendt, Frank R.Kolosov, DennisWeng, HongleiKoltes, JamesWeng, Mao-Lun (University of Texas)Komaromy, AndrasWeng, Mao-Lun (Westfield State University)Kominek, JacekWeng, YuhuiKomínek, PetrWenne, RomanKomissarov, AlekseyWessells, RobertKomuraiah, MyakalaWest, JamesKon, Mark A.Wevrick, RachelKondo, JiroWhipple, AmandaKondratova, MariaWhite, LaurenKong, YinfeiWhite, MichaelKonishi, HiroakiWhitehill, Justin G. A.Kononenko, Neonila V.Whitman, Barbara Y.Koornneef, MaartenWhittington, CarlKopjar, NevenkaWick, MacdonaldKoraimann, GüntherWidłak, WiesławaKorczeniewska, OlgaWiemels, Joseph L.Kordowitzki, PawełWiener, DominiqueKorec, EvzenWiley, VeronicaKorecky, JiriWilliams, Jessica A.Koren, AmnonWilliams, R. ScottKorsching, Sigrun IWilliamson, E. D.Korte, ArthurWilson, MaxKorzekwa, Anna.JWilson, Philippe B.Kosakyan, AnushWilton, SteveKosanovic, DjuroWimmers, KlausKosecka-Strojek, MajaWinkworth, RichardKosir, RokWit, Pierre DeKotov, Alexey A.Witarski, WojciechKottakota, ChandrasekharWitkowska-Piłaszewicz, OlgaKotyla, Przemyslaw J.Wittenburg, DörteKoudounas, KonstantinosWittmann, JürgenKovacs, Istvan A.Wnuk, AgnieszkaKovarik, AlesWoerner, August E.Kovinich, NikWolf, YuriKowalczyk, KrzysztofWollebo, Hassen S.Kowalewska-Łuczak, IngaWolstenholme, JenniferKowtharapu, Bhavani S.Won, Kyoung JaeKozlowski, LukaszWong, Boon SengKozma-Bognár, LászlóWong, LeeKozubowski, LukaszWong, Lee-Jun C.Kraaijeveld, KenWoo, Ho-HyungKrabbenhoft, Trevor J.Woo, Se JoonKrähenbühl, StephanWood, AndrewKraj, WojciechWoodrow, PasqualinaKramina, Tatiana E.Wook Kwon, SoonKranz, AngelaWoolley, ShernaeKrasikova, AllaWorth, James R. P.Krasileva, Ksenia V.Worthington, Margaret LeighKraska, PiotrWróblewska, JoannaKrasnov, AlekseiWrzaczek, MichaelKratochvíl, LukášWu, Alan H.Krauze, MagdalenaWu, Chen-ChiKrawczyk, BeataWu, HaoKrawczyk, PrzemekWu, KeqiangKrehenwinkel, HenrikWu, MengKrejci, LumirWu, RongxueKrell, TinoWu, Shu-BiaoKremer, Hannie P.H.BerryWu, YoujunKremer, NatachaWulff, BrandeKrenács, TiborWurdak, HeikoKrestinina, OlgaWurtzel, OmriKreutz, Rolf P.Wuyts, WimKrieg, Adam J.Wysocki, RobertKristensen, Lasse SommerXavier, AlencarKropp, MartinaXavier, CatarinaKrugman, TamarXiang, JimKruszewska, JoannaXiao, Shu-YuanKu, Cheol-ryongXie, WankunKubo, NakaoXu, HongKubo, TomohikoXu, HuichunKubota, AkaneXu, JingKuhlmann, MarkusXu, JintaoKuhlwilm, MartinXu, LingyangKühn, ChristinaXu, MaonianKuhnert, PeterXu, XiaojiangKuiper, RolandXu, XuanKulczyński, BartoszXynias, Ioannis N.Kulik, AnnaYadav, DhananjayKulkarni, AmitYadav, RachitaKulkarni, RaviYaeno, TakashiKumar, AnujYajima, MasanaoKumar, HirdeshYakimovich, ArturKumar, ManuYam, RitaKumar, RahulYamada, YasuhiroKumar, RajivYamaguchi, NobutoshiKumar, RohitYamaguchi, YoshihiroKumar, SantoshYamamoto, JunpeiKumar, SushilYamamura, TomohikoKumar-Sinha, ChandanYamanaka, YojiroKundrata, RobinYamasaki, MasanoriKurata, HiroyukiYamashino, TakafumiKurdyukov, SergeyYamashita, AkiraKurelac, IvanaYan, XiaKuroda, DaisukeYanda, MuraliKuroda, ShinnosukeYang, LiKuroi, YasuhiroYang, TaoKurpisz, MaciejYang, WangKusakabe, MakotoYang, XiaodongKushiro, TetsuoYang, XiaoheKutnjak, DenisYanling, LiaoKuzmanović, LjiljanaYann, AudicKuzmin, ElenaYao, ChaoqunKuźnicka, EwaYao, JianboKwack, KyuBumYaron, YuvalKwong, StephenYarullina, DinaKwun, Min JungYasuhara, TakaakiKyrgios, IoannisYavitt, JosephL. Pey, AngelYayan, JosefLa Mantia, IgnazioYazdanyar, AmirfarbodLabudda, MateuszYen, TimothyLacal, JesusYeung, Jacky T.Laciny, AliceYi, GibumLagali, PamelaYi, LiLai, XueleiYi, WangLakehal, AbdellahYi, ZhuLaliotis, George PYi-Fan, ChenLallemand, FraņcoisYing Kai, ChanLallena, María JoséYing, ShengLaloë, Jacques-OlivierYing, WangLamatsch, DunjaYingfang, ZhuLamb, DavidYinglin, XiaLamb, JanineYi-Qian, SunLampiasi, NadiaYi-Ting, LinLandi, VincenzoYochum, Gregory S.Landis, JacobYohay, KalebLange, Carol A.Yokota, HirokiLange, Philipp S.Yokoyama, ShigeruLanglade, NicolasYonekura, ShinichiLanglais, DavidYong, JiangLanning, NathanYong, Kar WeyLansdorp, Peter M.Yong-jie, XuLanza, Gaetano AntonioYonglun, LuoLanzaro, GregoryYonker, LaelLanzuolo, ChiaraYoon, Ki-JunLaplana, MarinaYoon, Sang-OhLargaespada, David A.York, DanielLargy, EricYoshida, KentaroLari, MartinaYoshida, KohtaLaribee, R. NicholasYoshiki, AtsushiLaRocque, JanYou, Hye JinLarraga, VicenteYou, Hyun JuLarson, AllanYou, Min KyoungLarson, Nicholas B.Young, Jason C.Lasagna, EmilianoYoung, JohnLasko, PaulYoung, Kendra A.Lattanzi, AnnalisaYu, ChenglongLau, BenjaminYu, FengqunLau, Kin H.Yuan, XuegangLaurence, DewachterYumoto, IsaoLaurencikiene, JurgaYurchenko, VyacheslavLauronen, JouniYzydorczyk, CatherineLaursen, Kristian BruunZaborowska, MagdalenaLawal, Hakeem O.Zaccai, MicheleLawrence, NicoleZadesenets, KiraLax, EladZádor, ErnőLayden, MichaelZadrag-Tecza, RenataLayman, Lawrence C.Zager, JordanLe Dévédec, SylviaZahan, MariusLe Pichon, Jean-BaptisteZaidi, Syed Shan-E-AliLeal, Ermelindo CarreiraZakrocka, IzabelaLeavesley, DavidZani, Bianca MariaLebedev, IgorZara, VincenzoLeblanc, OlivierZaritsky, AriehLebreton, SébastienZatsepin, TimofeiLecamwasam, AshaniZbigniew, MiszalskiLecellier, Charles-HenriZehner, RichardLeclère, LucasZeighami, YasharLederer, Carsten WernerZeller, Robert W.Lee, Daniel Y.Zemlyanskaya, Elena V.Lee, Hea-YoungZemmour, DavidLee, Hee JaeZevnik, BrankoLee, Jae-HoZhang, BaotongLee, JeehunZhang, ChiLee, Jong-EunZhang, FanLee, Jung MinZhang, JianhuiLee, Jung-HyunZhang, KeLee, KwanukZhang, PengLee, Kyung JunZhang, ShixiongLee, Min YoungZhang, WeiLee, OukseubZhang, XuekuiLee, Seung Hwan (Korea Research Institute of Bioscience and Biotechnology)Zhao, BaoyinLee, Seung Hwan (Seoul National University)Zhao, ChenLee, Sonny T.M.Zhao, DongyanLee, Soo InZhao, JinpingLee, Soong DeokZhao, WeixingLee, Wei-ChiehZhao, YuqiLee, Yang-SeokZheng, GuomaoLee, YiZhou, BangjunLegati, AndreaZhou, HangLehner, StefanieZhou, ZhenqiLehnert, Sigrid A.Zhu, Guo-ZhangLehti-Shiu, MelissaZhu, LiLei, FanZhu, Lihua JulieLei, TimZhu, WangshengLei, WeiZhu, XudongLeichty, AaronZhukov, Vladimir A.Leigh, Brittany A.Zieger, MartinLeisz, SandraZiko, LailaLeitão, AlexandreZilocchi, MaraLeitão, AnaZingaretti, Laura M.Lek, AngelaZinn, StevenLemonnier, LoicZinna, RobertLemos, Manuel C.Ziółkowska-Suchanek, IwonaLenart-Boroń, AnnaZok, TomaszLeng, RogerZollino, MarcellaLeonard, AlanZou, WeiLeonetti, ManuelZou, ZhongweiLeonhardt, RalfZrzavá, MagdaLe-Provost, FabienneZsolnai, AttilaLeroux, ChristineZsurka, GáborLesage, PascaleZubair, HaseebLeszczuk, AgataZubiaga, AnaLeszczynska, KatarzynaZubieta, ChloeLetarov, AndreyŻukowski, KacperLetko, AnnaŽulj Mihaljević, MajaLeu, Jiann-HorngŽunar, BojanLeung, ChristelleZwierzchowski, LechLevy, JulienZwinderman, Aeilko H.Lewinski, Nastassja A.


